# Late Graft Failure Due to Arterio-Venous Fistula in the Free Jejunal Graft Mesentery Following Total Pharyngo-Laryngo-Esophagectomy for Cervical Esophageal Cancer: A Case Report

**DOI:** 10.70352/scrj.cr.25-0147

**Published:** 2025-05-24

**Authors:** Koutarou Yamamoto, Tomoyuki Okumura, Takeshi Miwa, Yoshihisa Numata, Tatsuhiro Araki, Ayaka Itoh, Mina Fukasawa, Nana Kimura, Masakazu Nagamori, Kosuke Mori, Naoya Takeda, Tomohiro Minagawa, Kenta Sukegawa, Toru Watanabe, Katsuhisa Hirano, Isaya Hashimoto, Kazuto Shibuya, Isaku Yoshioka, Hideharu Abe, Toshihiko Satake, Noriko Okuno, Tsutomu Fujii

**Affiliations:** 1 Department of Surgery and Science, Faculty of Medicine, Academic Assembly, University of Toyama, Toyama, Toyama, Japan; 2 Office of Human Research Ethics, Faculty of Education and Research Promotion, Academic Assembly, University of Toyama, Toyama, Toyama, Japan; 3 Department of Otorhinolaryngology, Head & Neck Surgery, Faculty of Medicine, Academic Assembly, University of Toyama, Toyama, Toyama, Japan; 4 Department of Plastic, Reconstructive and Aesthetic Surgery, Toyama University Hospital, Toyama, Toyama, Japan; 5 Department of Pathology, Faculty of Medicine, Academic Assembly, University of Toyama, Toyama, Toyama, Japan

**Keywords:** esophageal cancer, total pharyngo-laryngo-esophagectomy (TPLE), free jejunal graft ischemia, refractory anastomotic fistula, antero-lateral thigh (ALT) flap

## Abstract

**INTRODUCTION:**

Insufficient blood supply to free jejunal grafts after total pharyngo-laryngo-esophagectomy (TPLE) occurs primarily due to failure of the vascular anastomosis, often resulting in rapid graft necrosis. This report details a case of ischemic enteritis caused by an arteriovenous fistula (AVF) in the mesentery of the free jejunal graft, resulting in chronic stenosis and total removal of the jejunal graft.

**CASE PRESENTATION:**

A 61-year-old woman diagnosed with squamous cell carcinoma of the cervical and thoracic esophagus underwent TPLE with gastric conduit and free jejunal graft reconstruction. The third jejunal artery and vein were anastomosed to the left transverse cervical artery and the internal jugular vein, respectively. On postoperative day (POD) 9, leakage was observed at the free jejunal-gastric anastomosis. The fistula healed with conservative treatment but a stenosis at the pharyngeal-jejunal anastomosis developed. Endoscopic observation after balloon dilation of the stenosis showed mucosal hemorrhage and ulcer scarring in the jejunal graft. A 3D reconstructed contrast-enhanced CT revealed the presence of an AVF in the free jejunal mesentery despite well-preserved blood flow across the vascular anastomosis. As no local inflammation was observed in the neck, and oral intake was sufficient after balloon dilatation, she was discharged from hospital. Seven months after surgery, she was admitted to our hospital due to obstruction of the pharyngeal-jejunal anastomosis with cutaneous fistula. Based on the disease course and endoscopic findings of the free jejunal graft, she was diagnosed with cutaneous fistula with scarring obstruction following chronic ischemic enteritis, considered difficult to heal with conservative treatment. Total removal of the free jejunum and reconstruction with an antero-lateral femoral thigh (ALT) flap was performed at 8 months after initial surgery. Oral intake was allowed on POD13, and she was discharged in good condition on POD30.

**CONCLUSIONS:**

We report here a rare case of late graft failure after TPLE due to chronic ischemia from an AVF in the mesentery of the free jejunal graft. Detailed assessment of mesenteric blood flow by 3D-constructed contrast-enhanced CT is useful and early removal of the ischemic jejunal graft is suggested.

## Abbreviations

AL
: anastomotic leakage
AVF
: arteriovenous fistula
CT
: computed tomography
PMMF
: pectoralis major myocutaneous flap
POD
: postoperative day
RF
: refractory anastomotic fistula

## INTRODUCTION

Free jejunal graft transplantation after total pharyngo-laryngo-esophagectomy (TPLE) for hypopharyngeal and esophageal cancer is a widely accepted standard reconstruction method, with a reported success rate of 92%–98%.^1–3)^ For cervical esophageal cancer, the incidence of graft failure in TPLE followed by reconstruction with a free jejunal graft is reported as greater than 3.0%,^4)^ with anastomosis-related vascular thrombosis noted as the most common cause.^5,6)^ Herein is reported a case of ischemic enteritis caused by an arteriovenous fistula (AVF) in the mesentery of the free jejunal graft resulting in a refractory anastomotic fistula and chronic stenosis, requiring repair with an antero-lateral femoral thigh (ALT) flap.

## CASE PRESENTATION

A 61-year-old woman was diagnosed with esophageal cancer; Ce (14–16 cm), 0-IIa, SCC, cT1b, cN0 cM0 cStageI, and Ut (20–29 cm), 0-IIb, SCC, cT0, cN0, cM0 cStage0, according to Japanese Classification of Esophageal Cancer 12th Edition.^7,8)^

In response to this diagnosis, TPLE was performed followed by reconstruction using gastric conduit and free jejunal graft. The third jejunal artery and vein were anastomosed to the left transverse cervical artery and the left internal jugular vein, respectively.

**Fig. 1:**
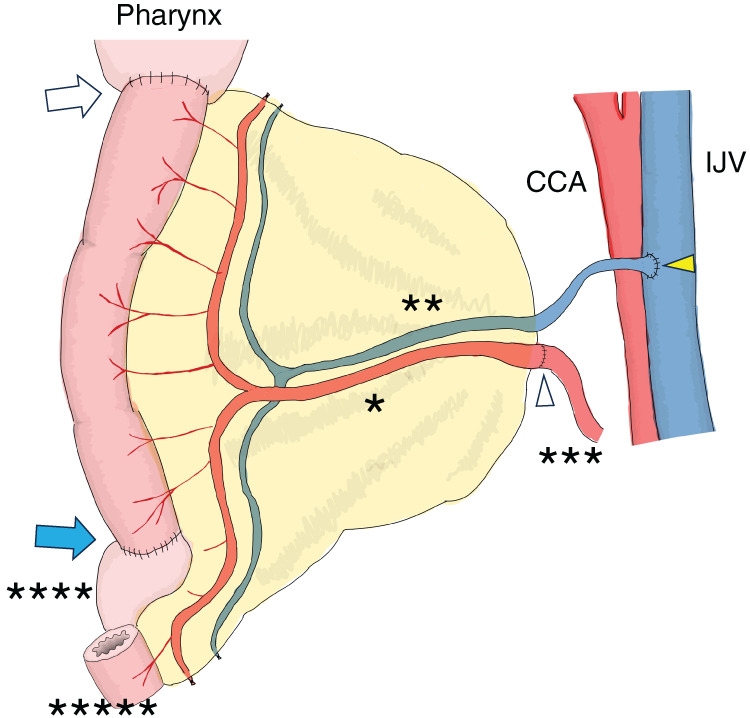
Schematic figure of reconstruction with free jejunal graft and gastric conduit. Single asterisk indicates the second jejunal artery. Double asterisk indicates the second jejunal vein. Triple asterisk indicates the left transverse cervical artery. Quadruple asterisk indicates the gastric conduit. Quintuple asterisk indicates the exteriorized monitor jejunum. The large white arrow indicates pharyngojejunal anastomosis. The blue arrow indicates gastro–jejunal anastomosis. The white arrowhead indicates vascular anastomosis between the second jejunal artery and the left transverse cervical artery. The yellow arrowhead indicates vascular anastomosis between the second jejunal vein and the left internal juggler vein. CCA, common carotid artery; IJV, internal juggler vein

Before wound closure, blood flow visualization using indocyanine green fluorescence confirmed sufficient blood supply in the vascular anastomosis, free jejunal graft, and gastric conduit. An exteriorized monitor jejunum and permanent tracheal stoma were created (**Fig. 1**).

**Fig. 2:**
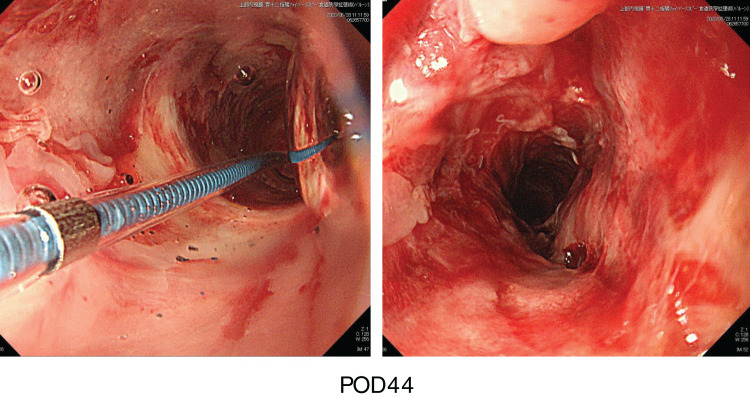
Endoscopic observation of the free jejunum at POD44. Balloon dilation of the anastomotic stenosis was performed.

The patient’s general postoperative condition was good and the monitor jejunum was removed on postoperative day (POD)5. On POD9, leakage at the free jejunum-gastric anastomosis was observed. Drainage of the subcutaneous abscess and the patient’s general condition were good, with no signs of free jejunal ischemia, so conservative treatment was continued. The fistula healed gradually, but after complete healing over a period of approximately 6 weeks, the patient suffered stenosis at the pharyngeal-jejunal anastomosis. Endoscopic observation after balloon dilation of the stenosis revealed mucosal hemorrhage and ulcer scarring. The mucosa was fragile, erosive and desquamated, indicating chronic change of ischemic enteritis (**Fig. 2**).

There was no sign of free jejunal necrosis. The patient’s dietary intake was good and had been a stable outpatient for several months.

**Fig. 3:**
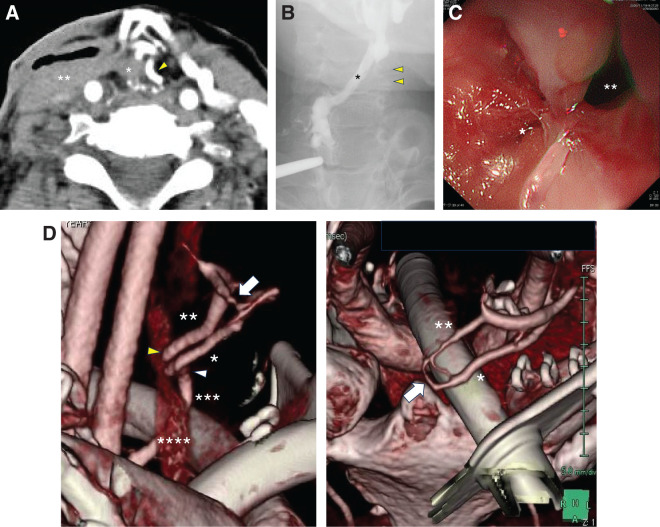
(**A**) Contrast-enhanced CT image at 7 months after surgery. The asterisk indicates the esophagus. The double asterisk indicates subcutaneous abscess. The yellow arrowhead indicates the third jejunal artery. (**B**) Fluoroscopy at 7 months after surgery. The asterisk indicates fistula at the pharyngealjejunal anastomosis with massive leakage. Yellow arrowheads indicate estimated location of the free jejunal graft. (**C**) Endoscopic observation revealed a complete obstruction due to scarring at the pharyngealjejunal anastomosis (asterisk) and a fistula opening on its side (double asterisk). (**D**) 3D reconstruction of a postoperative contrast-enhanced CT obtained on POD11. The single asterisk indicates the third jejunal artery. The double asterisk indicates the third jejunal vein. The triple asterisk indicates the left transverse cervical artery. The quadruple asterisk indicates the left internal jugular vein. The white arrowhead indicates vascular anastomosis between the second jejunal artery and the left transverse cervical artery. The yellow arrowhead indicates vascular anastomosis between the second jejunal vein and the left internal juggler vein. White arrows indicate the AVF in the free jejunal mesentery.

Seven months after surgery, the patient suffered from a right cervical subcutaneous abscess (**Fig. 3A**), which was incised, drained, and treated conservatively, resulting in a fistula at the pharyngeal-jejunal anastomosis with massive salivary leakage (**Fig. 3B**).

Upper gastrointestinal endoscopy revealed complete obstruction due to scarring at the pharyngeal-jejunal anastomosis and a fistula opening on its side (**Fig. 3C**). Detailed re-observation with 3D reconstruction of a post-operative contrast-enhanced CT obtained on POD11 for leak assessment, which was not reconstructed in 3D at the time, showed that arterial and venous blood flow across the vascular anastomosis was well preserved, but there was an AVF in the free jejunal mesentery (**Fig. 3D**).

Therefore, the patient was diagnosed with refractory cutaneous fistula with scarring obstruction after chronic ischemic enteritis of the free jejunum and considered difficult to heal with conservative treatment.

Eight months after initial surgery, the patient underwent total removal of the free jejunum, reconstruction with an ALT flap and segmental skin grafting.

**Fig. 4:**
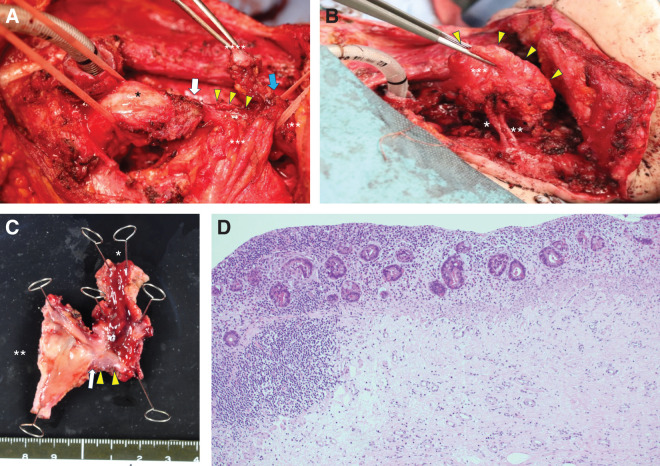
The operative findings of total removal of the free jejunal graft. (**A**) The free jejunum was atrophic and scarred. Scheme of the operative view is on the right. Single asterisk indicates the gastric conduit. Double asterisk indicates the pharynx. Triple asterisk indicates the free jejunal mesentery. Quadruple asterisk indicates the pharyngo-cutaneous fistula. Yellow arrowheads indicate the free jejunum with atrophic stenosis. The white arrow indicates the gastrojejunal anastomosis. The blue arrow indicates the pharyngojejunal anastomosis. (**B**) The free jejunal graft was removed by dissecting along the free jejunal wall while preserving the mesentery. Single asterisk indicates the third jejunal artery. Double asterisk indicates the third jejunal vein. These vessels were skeletonized in preparation for vascular anastomosis. Triple asterisk indicates the preserved jejunal mesentery. Yellow arrowheads indicate the edge of the jejunal mesentery after removing free jejunum. (**C**) A gross specimen of the removed free jejunal graft. Single asterisk indicates the oral wedge of the free jejunal graft (pharyngeal side). Double asterisk indicates the anal wedge of the removed specimen (gastric conduit side). The white arrow indicates the gastro–jejunal anastomosis. Yellow arrowheads indicate the removed free jejunum with scarred stenosis. (**D**) A histologic image of the removed free jejunal graft. There was extensive epithelial atrophy and thinning of the mucosa-specific layer, with the remaining glandular ducts shrinking and showing severe fibrosis in the submucosal layer (×200).

The free jejunum was atrophic and scarred, with a diameter of approximately 7 mm and length of approximately 30 mm (**Fig. 4A**). Blood flow across the vascular anastomosis in the jejunal mesentery was good and the arterio-venous shunt was strongly palpated with thrill. The specimen was removed by dissecting along the free jejunal wall while preserving the mesentery (**Fig. 4B**).

A gross specimen of the removed free jejunum showed atrophy and hard scarring, with a high degree of stenosis of the lumen (**Fig. 4C**). Histologically, there was extensive epithelial atrophy and thinning of the mucosa-specific layer, with the remaining glandular ducts shrinking and showing severe fibrosis in the submucosal layer (**Fig. 4D**). These findings suggested ischemic changes, but no thrombosis was observed.

Postoperative fluoroscopy on POD10 showed a smooth passage at the repaired site with oral intake started on POD13. The patient was discharged with good physical condition on POD30. Four years have passed since the repair operation with good general condition while undergoing endoscopic balloon dilation for occasional stenosis of the pharyngeal-ALT flap anastomosis.

## DISCUSSION

Herein is reported a rare case of late graft failure after TPLE due to chronic ischemia of the free jejunal graft caused by an AVF in the mesentery. The most commonly reported cause of graft failure in TPLE with free jejunal graft is anastomosis related vascular thrombosis.^5,6)^ In addition to thrombosis, local physical factors such as compressive pressure from the hematoma and the effect of infection of the vascular pedicle are other causes of microsurgical ischemia.^9)^ There is also a report of non-occlusive mesenteric ischemia (NOMI) of a free jejunal flap, presumably due to insufficient blood flow from systemic pre-shock during the early postoperative period.^9)^ However, to date, there are no reports of free jejunal flap ischemia caused by mesenteric AVF. It is also noteworthy that, unlike the rapid necrosis progression observed in NOMI, the scarring stenosis of the free jejunal graft observed here developed over a chronic course of 6 months, eventually requiring total graft removal, with a course similar to that of ischemic enteritis.

Ischemic enteritis occurs from insufficient arterial inflow to the small intestine without apparent obstruction of the main vessels, commonly in elderly people with thrombotic conditions such as hypertension, ischemic heart disease, arrhythmia, cerebral infarction, or diabetes.^10)^ Unlike ischemic colitis, most ischemic enteritis is characterized by irreversible ischemia that progresses in a chronic phase causing small intestinal stenosis, requiring surgical resection several months after symptom onset.^10)^ Endoscopically, circumferential ulcerations and afferent stenosis are associated with ischemic enteritis. Additionally, histological features of ischemic enteritis are ulcerations deeper than the submucosal layer, with marked fibrosis of the submucosa,^11)^ findings similar with our present case.

Superior mesenteric AVF are very rare, with no reports for peripheral areas close to the small bowel wall as in the present case. The AVF is an abnormal connection between arteries and veins bypassing the capillary bed, decreasing arterial flow and increasing venous pressure in the tissue beyond the fistula, usually resulting in bowel ischemia secondary to the steal phenomenon.^12)^ This may occur congenitally but often arises after abdominal trauma or iatrogenic injury during gastrointestinal surgery, typically as a result of ligation of both artery and vein together or ligation in the mesentery without localization of the bleeding point.^12)^

It is unclear whether the AVF in this case was present prior to the surgery as the routine preoperative contrast-enhanced CT was not of sufficient quality to allow reconstruction of the small intestinal mesentery peripheral vascular structures. Although the patient had no history of abdominal trauma, either cause is possible. The free jejunum was carefully handled to protect it during surgery, and no vascular ligation or hemostatic procedure was performed in the free jejunal mesentery. It is also reported that AVF often develops several years after trauma or surgery,^12)^ which contrasts to this case with AVF detected by high-resolution CT recorded on POD11. However, intraoperative blood flow visualization using indocyanine green fluorescence confirmed sufficient blood supply to the vascular anastomosis and free jejunal graft, and no surgeon noted the presence of an AVF in the jejunal graft mesentery. In either case, it is possible that rapid development of an initially micro AVF after free jejunal graft reconstruction with progression of the steel phenomenon was due to limited inflow and outflow to only the anastomotic vessels.

Superior mesenteric AVFs generally occur between the superior mesenteric artery and vein, and are associated with risks of abdominal angina, intestinal bleeding, and portal hypertension. Therefore, endovascular embolization is considered as a treatment option.^13)^ However, there are no reports of endovascular treatment of AVFs in the peripheral part of the mesentery. Additionally, embolizing the AVF in the jejunal graft beyond the anastomosis between the third jejunal artery and the left transverse cervical artery may be challenging. There is also a risk of graft necrosis as a complication of endovascular embolization.^13)^ Together with the possible progression of jejunal graft stenosis, similar to the course of ischemic enteritis, early graft removal is considered to be the most effective treatment. In the present case, the AVF was found retrospectively by constructing 3D-CT images in preparation for graft removal surgery, resulting in a prolonged period of conservative treatment. Therefore, a detailed examination of the blood flow, not only at the vascular anastomosis but also at the peripheral vessels in the mesentery, is necessary when ischemic changes of the free jejunal graft are observed.

Several monitoring techniques are available to evaluate free jejunal graft viability, including exteriorization of a jejunal segment, implantable Doppler probe, and external color Doppler ultrasound.^14,15)^ These reports assessed free jejunum viability during earlier postoperative periods until PODs 7 to 10. We also monitored the viability of the free jejunal graft by exteriorized jejunum and percutaneous color Doppler probe, but the findings were not indicative of mesenteric AVF. Therefore, it is suggested that 3D-constructed contrast-enhanced CT is useful for AVF detection in the mesentery of the free jejunal graft.

## CONCLUSION

We report a rare case of late graft failure after TPLE due to chronic ischemia from an AVF in the mesentery of the free jejunal graft. Detailed assessment of mesenteric blood flow by 3D-constructed contrast-enhanced CT is useful and early removal of the ischemic jejunal graft is suggested.

## DECLARATIONS

### Funding

This work was partly supported by JSPS KAKENHI, Grant No. 24K11844.

### Authors’ contributions

K. Yamamoto and T. Okumura produced the case report conception and design, and wrote the main manuscript body.

T Okumura, T Miwa, T Watanabe, H Abe, and T Satake obtained informed consent and performed surgery.

K Yamamoto, T Okumura, T Miwa, Y Numata, A Itoh, M Fukasawa, N Kimura, M Nagamori, T Araki, K Mori, N Takeda, T Minagawa, K Sukegawa, T Watanabe, K Hirano, I Hashimoto, K Shibuya, I Yoshioka, H Abe, T Satake, and T Fujii participated in the patients’ care and critically reviewed the manuscript.

N Okuno participated in pathological diagnosis and critically reviewed the manuscript.

All authors have read and approved the manuscript.

### Availability of data and materials

The datasets of this case report are available from the corresponding author upon reasonable request.

### Ethics approval and consent to participate

This work does not require ethical considerations or approval. Informed consent to participate in this study was obtained from the patient.

### Consent for publication

Informed consent was obtained from the patient for the publication of this case report and accompanying images.

### Competing interests

The authors have no related conflicts of interest to declare.
